# Brain metastases in patients with neuroendocrine neoplasms: risk factors and outcome

**DOI:** 10.1186/s12885-019-5559-7

**Published:** 2019-04-16

**Authors:** Sebastian Krug, Freya Teupe, Patrick Michl, Thomas M. Gress, Anja Rinke

**Affiliations:** 1 Clinic for Internal Medicine I, Martin-Luther University Halle/Wittenberg, Ernst-Grube-Straße 40, 06120 Halle, Germany; 2 Department of Gastroenterology and Endocrinology, University Hospital Marburg, Baldinger Strasse, 35043 Marburg, Germany

**Keywords:** Brain metastasis, Neuroendocrine tumor, Neuroendocrine carcinoma, Whole brain radiotherapy, Prognosis

## Abstract

**Background:**

Brain metastases (BM) are rarely reported in patients with neuroendocrine carcinoma (NEC) of non-lung origin and neuroendocrine tumors (NET) of the gastroenteropancreatic (GEP) or bronchopulmonary system. However, symptomatic brain metastases are associated with dismal prognosis, so early detection and treatment could be advisable.

**Methods:**

We retrospectively analyzed 51 patients with GEP-NEN and bronchopulmonary NEN excluding small cell lung cancer. All patients were treated at the University Hospital Marburg and Halle (Saale) between 2000 and 2017. The median overall survival (mOS) and mOS after diagnosis of brain metastases (BM) were calculated using Kaplan-Meier analysis. Risk factors for poor prognosis were evaluated using univariate and multivariate Cox regression method.

**Results:**

Overall, 51 patients with a median age of 58 years presented BM. Lung (*n* = 23, 45.1%) was the most frequent primary localization. Most patients had NEC (*n* = 31, 60.8%), including 26 carcinomas (51%) with Ki-67 indices > 55%. Singular BM were present in 16 patients (31.4%), but 21 patients (41.2%) had multiple lesions. Overall, the median period from first diagnosis of the tumor disease up to diagnosis of brain metastasis was 5.0 months. Palliative radiation was the most common therapy (*n* = 31, 60.8%). Median OS after initial diagnosis and diagnosis of BM was 23.0 and 11.0 months, respectively. Univariate and multivariate analysis for prognostic indicators depicted differentiation (NEC HR 4.2, 95% CI 1.1–16.1) and age (≥60 HR 3.0, 95% CI 1.2–7.5) as markers for poor outcome.

**Conclusions:**

Overall, the risk for symptomatic brain metastases is low in GEP-NEN and bronchopulmonary NEN patients. Age above 60 and poor tumor differentiation may deteriorate the overall survival. Therefore, screening for brain metastases could be advisable in NEC patients.

## Background

Brain metastases are the most common intracranial neoplasm in adults. They often originate from lung cancer, breast cancer or melanoma, but also other malignancies like renal cancer, colorectal cancer and ovarian cancer are increasingly associated with brain metastases [1]. Nearly 20% of the patients with small cell lung carcinoma (SCLC) demonstrate brain metastases at initial diagnosis and about half of the patients develop brain metastases during follow up [2]. In contrast, brain metastases are rarely reported in patients with neuroendocrine carcinoma of non-lung origin and neuroendocrine tumors (NETs) of the gastroenteropancreatic or bronch-opulmonary system. In the Spanish and German NET Registries 4 of 837 (0.5%) and 12 of 2358 (0.5%) patients with brain metastases are documented [3, 4]. The estimated incidence in NETs is 1.5–5% [5].

General screening for brain metastases is not recommended in NET and non-lung NEC patients. Whether or not prophylactic brain irradiation in limited disease NEC of gastroenteropancreatic origin could result in better prognosis like in SCLC [6] is unknown. Symptomatic brain metastases are often associated with a dismal prognosis, so early detection and treatment could be advisable.

The aim of our study is therefore to analyze frequency, origin, treatment and outcome of brain metastases in two single centre cohorts of NET and NEC patients with long follow up.

## Methods

In 1998, we established a database for patients with NEN of different origins including bronchopulmonal NETs and NECs of gastroenteropancreatic or unknown origin, who presented at our university hospital in Marburg for diagnosis and/or treatment of the tumor disease. Patients with small cell lung cancer were not included in this database. First data were retrospectively documented and patients then prospectively followed. Collected data included clinic-pathological features and date of death or date of last contact. Collection, storage, and evaluation of patient related information in our NEN database was done with patient informed consent and with the approval of the local ethics committee at the University of Marburg. This study was conducted in accordance with the Declaration of Helsinki. In Halle (Saale), we used a retrospectively created database, in which all patients with neuroendocrine tumors and carcinomas were included. All patients with neuroendocrine neoplasm and brain metastases were included. Patients with SCLC were excluded. The large-cell lung NECs are not completely recorded in our database. Patients with large-cell lung NEC and sufficient data documentation were included. Our hypothesis was that patients with brain metastases had a worse survival and preferably patients with lung-NEN were affected by brain metastases.

No routine cerebral imaging was performed. Patients with various CNS complaints, including seizures, vertigo, motoric and sensoric deficits and headaches were examined. In some clinical situations it was also the sole individual medical decision that led to the performance of brain imaging.

Statistical analysis was performed using IBM SPSS Statistics. Kaplan-Meier analyses of overall survival and survival since diagnosis of brain metastases were generated. We used the log-rank test to detect statistically significant differences between groups. Significance was defined as *p* < 0.05. Univariate and multivariate analysis was performed using Cox Proportional Hazards Regression.

## Results

### Patient and tumor characteristics

In total, we identified 51 patients with brain metastases out of our patient records (Table 1). The mean age at the time of diagnosis was 56 years (range 27–86). This group included 25 women (49%) and 26 men (51%). The majority of patients 48/51 (94.1%) had nonfunctioning tumors. Hormonal syndromes included Zollinger-Ellison Syndrome (*n* = 2) and Cushing syndrome (ectopic ACTH; *n* = 1). Seventeen of 51 patients (33.3%) had well-differentiated tumors, 31 patients (60.8%) had poorly differentiated neuroendocrine carcinoma and in nine patient (17.6%) tumor differentiation was not documented. In 26 patients (51%) Ki-67 index was 55% or higher. Most patients had “foregut” primaries with bronchial/lung (*n* = 23; 45.1%) and pancreas (*n* = 9; 17.6%) being the most prevalent ones. Other primaries were located gastrointestinal (*n* = 5; 9.8%) and in cervix/ovary (n = 2; 3.9%). In 12 patients, primary localization was unknown during the initial diagnosis and treatment course (Table 1). No valid information can be given about the incidence of BM. Based on the Marburg cohort 1,6% of patients presented with brain metastases (16 out of 975 patients screened).

**Table 1:** Summary of patient characteristics

Characteristics	Number of all patients (%)	All NET patients (%)	All NEC patients (%)	Long-term survivors (%)	Metachrone BM (%)
Total	51	17 (33)	31 (61)	6 (11.7)	20 (39)
Mean age at first diagnosis (years)(range)	56 (27–86)	55 (27–78)	55 (27–86)	54 (35–66)	51 (30–69)
Mean age at diagnosis of brain metastases (years)(range)	58 (27–86)	56 (27–58)	56 (27–86)	58 (45–74)	55 (32–73)
Primary tumor localization					
lung	23 (45.1)	6 (35.3)	17 (54.8)	4 (67)	4 (20)
CUP	11 (21.6)	4 (23.5)	5 (9.8)		3 (15)
pancreas	9 (17.6)	4 (23.5)	4 (12.9)	1 (16.5)	9 (45)
gastrointestinal tract	6 (11.8)	2 (11.8)	4 (12.9)		2 (10)
cervix/ovary	2 (3.9)	1 (5.9)	1 (3.2)	1 (16.5)	2 (10)
Gender					
male	25 (49.0)	5 (29.4)	18 (58.1)	0	6 (30)
female	26 (51.0)	12 (70.6)	13 (41.9)	6 (100)	14 (70)
Histology WHO 2010					
well/moderate					
differentiated	17 (33.3)	17 (100)	0	5 (83.5)	7 (35)
poorly differentiated	31 (60.8)		31 (100)	1 (16.5)	11 (55)
unknown	3 (5.9)				2 (10)
Ki-67 index					
G1 (≤2%)	3 (5.9)	3 (17.6)	0	0	3 (15)
G2 (3–20%)	14 (27.5)	14 (82.4)	0	4 (67)	4 (20)
G3 (> 20%)	31 (60.8)		31 (100)	1 (16.5)	11 (55)
< 55%	16 (31.4)		13 (41.9)	1 (16.5)	6 (30)
> 55%	26 (51.0)		16 (51.6)	0	5 (25)
unknown	9 (17.6)		2 (6.5)	1 (16.5)	3 (15)
Functionality					
non-functioning	48 (94.1)	15 (89.2)	30 (96.8)	4 (67)	18 (90)
gastrinoma	2 (3.9)	1 (5.9)	1 (3.2)	1 (16.5)	2 (10)
ectopic ACTH syndrome	1 (1.9)	1 (5.9)		1 (16.5)	
Sites of other metastases					
Liver	31 (60.8)	13 (76.5)	17 (54.8)	3 (50)	12 (60)
lymph nodes	28 (54.9)	8 (47.1)	20 (64.5)	4 (67)	12 (60)
bone	23 (45.1)	10 (29.4)	12 (38.7)	1 (16.5)	9 (45)
lung	14 (27.5)	5 (14.7)	9 (29.0)	2 (33)	4 (20)
adrenal	5 (9.8)		5 (16.1)	0	1 (5)
none	2 (3.9)		2 (6.5)	0	0
other	18 (35.3)	6 (35.3)	12 (38.7)	4 (67)	6 (30)
Number of brain metastases					
1–2	22 (43.1)	9 (52.9)	13 (41.9)	1 (16.5)	9 (45)
≥ 3	21 (41.2)	5 (29.4)	14 (45.2)	5 (83.5)	8 (40)
unknown	8 (15.7)	3 (17.7)	2 (6.5)		3 (15)
Therapy of brain metastases					
radiation	31 (60.8)	8 (47.1)	21 (60.8)	4 (67)	10 (50)
none	19 (37.3)	9 (52.9)	9 (29.0)	2 (33)	7 (35)
resection	7 (13.7)	1 (5.9)	6 (19.4)	1 (16.5)	4 (20)
temozolomide-based CTx	7 (13.7)	4 (23.5)	2 (6.5)	2 (33)	4 (20)

Abbreviations: *NET* neuroendocrine tumors; *NEC* neuroendocrine carcinomas; *BM* brain metastases; *CUP* carcinoma of unknown primary; *CTx* chemotherapy

### Latency first diagnosis – diagnosis brain metastases

Median time from initial diagnosis of neuroendocrine neoplasia until diagnosis of brain metastases was 5 months (range 0–144 months). In two patients, a seizure due to brain metastasis was the first symptom of the tumor disease.

### Tumor stage at diagnosis and localization of distant metastases

In 49 of the 51 patients (96.1%), distant metastases beyond BM were present at diagnosis (stage IV). All but 11 patients developed multiple localizations (more than 2) of distant metastases. The most frequent site was liver (31/51; 60.8%), followed by lymph nodes (28/51; 54.9%) and bone metastases (23/51; 45.1%). Other localizations included lung (14/51; 27.5%), adrenal (5/51; 9.8%), peritoneum (4/51; 7.8%), subcutaneous metastasis (3/51; 1.9%), meningeosis (1/51; 1.9%), spleen (1/51; 1.9%), ovaries (1/59; 1.9%) and renal (1/51; 1.9%).

### Treatment and outcome data

**Fig. 1:**
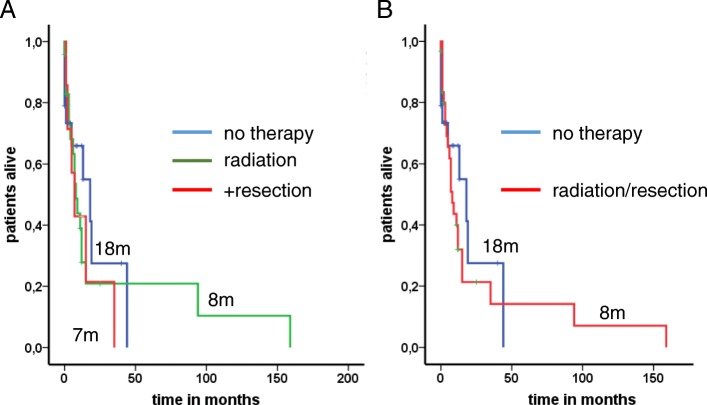
Median overall survival times with brain metastases under therapy. There were no significant differences in patients with BM treated with radiation, surgery plus radiation or observation (8 vs. 7 vs. 18 months; *P* = 0.72) **(a)**. When dividing these patients in an observation and therapeutic arm similar results were obtained (18 vs. 8 months; *P* = 0.46) **(b)**

When BM were confirmed radiologically, evaluation of comorbidities and patient characteristics was performed in order to guide the optimal treatment approach. Since time from onset of disease to BM was short in median (5 months), most patients suffered from an aggressive and progressive tumor disease, thus all patients received systemic therapy beyond local cerebral treatment. Whole brain radiation was performed in 31 patients (60.8%), whereas only a minority of seven patients were eligible for resection (Table 1). Chemotherapy with alkylating agents such as temozolomide, which might bypass the blood brain barrier were used in seven patients. All other patients received steroids and symptomatic treatment beside systemic chemotherapy. In respect to the different therapeutic approaches for BM, no differences for the OS have been obtained (Fig. 1a and b). After BM were diagnosed median overall survival (mOS) was 11 months (95% CI 5.3–16.7). The 2-year survival rate was calculated with 12.7%. Only two patients with well-differentiated lung-NETs presented a long-term survival of more than 5 years (94 and 159 months). Additionally, assessment of risk factors for poor survival were done in a univariate and multivariate fashion including gender, localization of the primary, differentiation, Ki-67 cut-off 55%, age, metastatic pattern and number of brain metastases (Table 2). Univariate analysis revealed male gender (HR 2.7; 95% CI 1.2–5.9), age ≥ 60 (HR 2.1; 95% CI 1.0–4.3), and differentiation (HR 2.1; 95% CI 1.0–4.3) as risk factors. Further evaluation showed poor differentiation (HR 4.2; 95% CI 1.1–16.1) and higher age (≥60) (HR 3.0; 95% CI 1.2–7.5) as independent marker for poor prognosis in multivariate tests (Table 2).

**Table 2:** Univariate and multivariate analysis for prognostic indicators

Variable	univariate			multivariate		
	HR	95% CI	*p*-value	HR	95% CI	*p*-value
Gender						
female	1			1		
male	2.7	1.2–5.9	*0.013	1.3	0.5–3.8	0.57
Localization						
lung	1			1		
non-lung	1.1	0.6–2.3	0.74	1.4	0.6–3.4	0.46
Grade						
NET	1			1		
NEC	2.7	1.2–6.2	*0.022	4.2	1.1–16.1	*0.038
Ki-67						
< 55%	1			1		
> 55%	1.5	0.6–3.4	0.39	0.9	0.3–2.8	0.94
Age						
< 60	1			1		
≥ 60	2.1	1.0–4.3	*0.041	3.0	1.2–7.5	*0.016
Metastases						
LN +/− liver	1			1		
LN +/− liver +	0.5	0.2–1.2	0.10	1.5	0.5–4.6	0.47
other						
No. of BM						
≤ 2	1			1		
> 2	0.9	0.4–2.0	0.79	0.7	0.3–1.8	0.51

Abbreviations: *NET* neuroendocrine tumors; *NEC* neuroendocrine carcinomas; *BM* brain metastases; *HR* hazard ratio; *CI* confidence interval; *LN* lymph nodes

*P<0.05

### Distinctions between G1/G2 (NET) and G3 (NEC) neoplasms

**Fig. 2:**
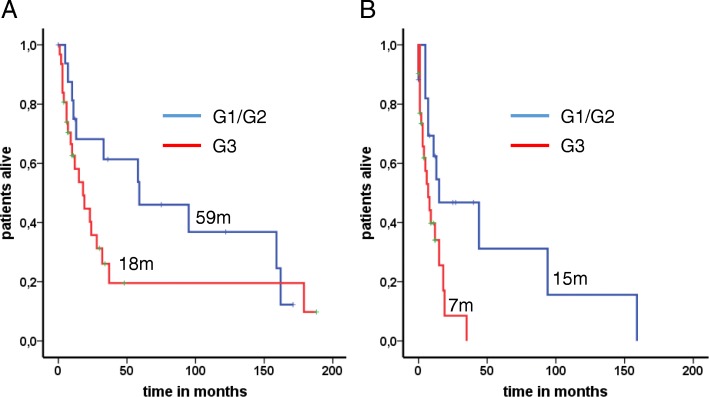
Median overall survival depends on grading. G1/G2 tumors presented a non-significant longer median overall survival in comparison to G3 tumors (59 vs. 18 months; *P* = 0.12) **(a)**. After validation of BM there was a significant distribution between both entities in favour of the well-differentiated neoplasms (15 vs. 7 months; *P* = 0.015) **(b)**

**Fig. 3:**
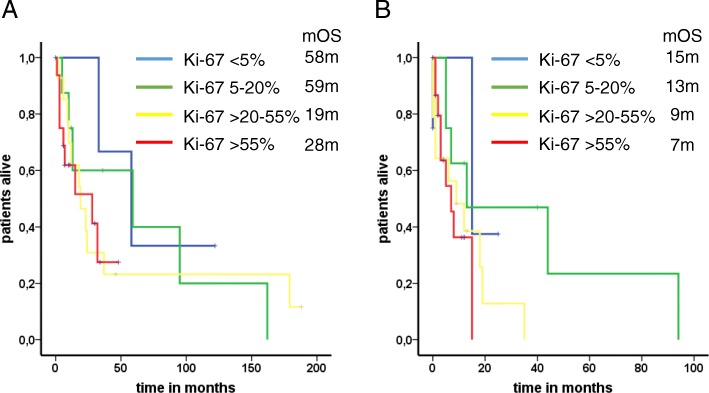
Median overall survival based on the proliferation rate. Neuroendocrine neoplasms were distributed based on their proliferation rate: < 5%, 5–20%, > 20–55 and > 55%. Regarding median overall survival from initial diagnosis, there was no significant result after within the G3 neoplasms (Ki-67 > 20–55% vs. > 55%, 19 vs. 28 months; *P* = 0.91) **(a)**. However, after BM detection the proliferation rate was associated with a worsened outcome (15, 13, 9 and 7 months), although statistical significance was not reached (*P* = 0.14) **(b)**

In Table 1 differences between G1/G2 and G3 neoplasms are depicted. Whereas mean age at initial diagnosis and BM detection was similar (55 years and 56 years in both groups), the gender distribution showed a significant trend towards more female patients in the G1/G2 cohort (G1/G2 vs. G3; 70.6% vs. 41.9%; *P* = 0.075). Furthermore, the proportion of patients with lung origin differed (G1/G2 vs. G3; 35.3% vs. 54.8%) non-significantly (*P* = 0.23) between both groups. Concerning the metastastic spread it was apparent, that adrenal metastases have been only detected in the G3 group in patients with lung primaries (*n* = 5; 16.1%). Although numeric differences were seen in the distribution of brain metastases (G1/G2 vs. G3; BM ≥ 3 29.4% vs. 45.2%), no statistically significant results were reached. The latency times between both entities were quite similar (4 months vs. 3 months). Analysis of mOS G1/G2 and G3 neoplasms after initial diagnosis (59 months vs. 18 months; *P* = 0.12) and after diagnosis of BM (15 months vs. 7 months; *P* = 0.015) confirmed an improved outcome for those with well-differentiated tumors (Fig. 2a and b). When assessing the outcome grouped by distinct Ki-67 indices (< 5%, 5–20%, > 20–55 and > 55%), a linear survival impairment correlated with an increased proliferation rate after BM occurred (mOS: 15, 13, 9 and 7 months) (Fig. 3b). However, the proliferation rate was unable to significantly discriminate the overall prognosis for those patients with G3 neoplasms (Ki67 20–55% vs. > 55%: 28 months vs. 19 months).

### Long-term survivors and patients with metachronous BM

Patients with survival times more than 24 months after diagnosis of brain metastases (BM) and patients with metachronous BM (at least 12 months after initial diagnosis) were evaluated separately. In the first group, six female patients met the inclusion criteria. Primary tumor localization was lung (*n* = 4; 67%), pancreas and ovary (both *n* = 1; 16.5%). Except one patient (G3 tumor), all presented G2 well differentiated tumors. Median latency before BM detection and median survival with BM was 4 and 42 months, respectively. Three patients with BM were diagnosed within 4 months, the others after at least 8 years. Four patients underwent radiotherapy, one patient in combination with local resection. The patient with combined treatment was the only patient suffering from a grade 3 neoplasm who survived more than 24 months after diagnosis of BM.

The metachronously metastasized cohort included 20 patients (median latency 33 months) Pancreas was the leading origin for primary localization with 45% (*n* = 9), followed by lung (*n* = 4; 20%) and unknown origin (*n* = 3; 15%). Morphologically, in seven and in 11 patients well- and poorly differentiated neoplasms appeared, respectively. Before BM occurred all patients suffered from further tumor manifestations in lymph nodes (*n* = 12; 60%), liver (*n* = 12; 60%) and bones (*n* = 9; 45%). The distribution of BM was similar to the entire cohort. Half of all were irradiated, four resected, four received temozolomide and seven had no additional approach beyond their ongoing systemic therapy. Overall, in patients with metachronous BM the mOS reached 12 months (CI 95% 4.2–19.8).

## Discussion

Metastatic disease is a major prognostic factor in neuroendocrine neoplasms in addition to differentiation and proliferation rate [7]. Besides common distant manifestations including lymph node, liver and bone metastases, brain metastases (BM) in NET are rare with an estimated incidence < 5% [8, 9]. Based on our own results, the incidence for brain metastases is 1–2%. However, the current data suggest a worse prognosis in patients with BM [9, 10]. Known factors influencing overall survival are primary tumor localization and TNM classification [11, 12]. Additionally, own previously published data presented impaired survival rates for patients with bone metastases [13]. However, median overall survival from the initial diagnosis of stage IV GEP-NET is about 90 months, which is considerably longer than in our cohort of patients with BM [13]. In general, for BM most data are available in bronchopulmonary NEN. Particularly, in large-cell NEC of the lung BM are frequently detected in up to 50% of cases [14]. Only small retrospectively collected case series or individual reports of BM in NET patients have been published. In this light, our data provide important clinico-pathological information of patients with BM. Our cohort included more than 50 patients, divided into G1/G2 vs. G3 neoplasms, long-term survivors and metachronously metastasized patients. In our series, the median overall survival time was 11 months, which is in line with other retrospective series with survival times of 6–10 months [9, 10, 15, 16]. However, comparison of outcome in published retrospective series is hampered because of the different composition of cohorts. The shorter mOS of only 8.1 months after diagnosis of BM in the cohort of Akimoto et al. may be explained by a higher proportion of patients with NEC [10]. Nevertheless, the mOS of 11 months in our series also compares favourably to the 10 months mOS reported by Hlatky et al. who included 24 “carcinoid patients” - a term usually used for well-differentiated tumors although differentiation or grade is not clearly mentioned in this paper [9].

In almost all cases, the presence of BM was a characteristic of a systemic dissemination and disease progression. In the only two cases without any other distant metastases the primary was located in the lung. Interestingly, although the majority of patients included in our databases suffer from gastroenteropancreatic NEN, in our cohort of patients with BM the lung was the most frequent primary tumour localization, which is in accordance with the other retrospective series [9, 10]. In comparison to recently published studies the mean age of our group was rather young (< 60 years) and consistent between NET and NEC [10, 16]. Whereas in the whole cohort both genders were similarly affected, female patients dominated the long-term survivors and metachronous BM group. The median latency time for BM was rather short in our series (5 months; range 0–144 months) and interestingly, no significant difference within the grading has been observed. Hlatky et al. and Akimoto et al. reported median times from initial diagnosis to BM detection of 18 and 12.8 months, respectively [9, 10], the latter, despite the inclusion of a significant proportion of large-cell and small-cell lung carcinomas. Thus, we believe, cerebral imaging has been used early in our cohort to detect or rule out disease progression in comparison to other studies. However, no routine cerebral imaging was implemented in the metastatic assessment of our patients. When evaluating risk factors for impaired prognosis, the differentiation based on the grading distributed the patients’ course significantly [17, 18]. This was also observed in our cohort and noteworthy, since G1/G2 and G3 neoplasms presented similar latency times for BM, these results reflected the diverse tumor biology and progression. Further stratification of G3 graded tumors with respect to an arbitrary proliferation activity of 55% as previously published by Sorbye, however, did not contribute to precise information [19].

Beyond tumor-related characteristics, the optimal treatment for BM in patients with neuroendocrine neoplasms needs to be clarified. Obviously, there are no prospective and randomized data aiming at this issue. In general, several treatment modalities are available using surgery, stereotactic radiosurgery, whole brain radiotherapy (WBRT), or chemotherapy as single therapies or in combination. Data from extracranial metastatic surgery, in particular, in liver approaches of patients with small bowel and pancreatic primaries favour a radical treatment and resection in patients with well-differentiated neuroendocrine tumors [20, 21]. In our series, none of the given treatment options was clearly superior. Several articles described a survival advantage when surgery of BM was followed by adjuvant WBRT [9, 15, 16, 22, 23]. The authors concluded that surgery combined with WBRT might be feasible in patients with good preoperative performance status and a solitary BM. In our cohort the only long term survivor with grade 3 tumour also received the combination of resection and radiotherapy. For patients with small cell lung cancer and BM, stereotactic radiosurgery combined with WBRT has generally been recommended as first choice treatment [24]. In other neuroendocrine neoplasms, no data on the dual therapy are available. However, Kawabe and co-workers presented a study using stereotactic radiosurgery alone in patients with BM and large cell NEC of the lung. The tumor control rate was 86% after 12 months [16]. Additionally, the neurocognitive status was nearly unaffected in these patients and that is a major benefit in contrast to the WBRT. In some patients of our cohort, chemotherapy with temozolomide was administered. The benefit of temozolomide has been shown in well-differentiated pancreatic neoplasms, in NEC in combination with capecitabine or as monotherapy in lung NEN [7, 25, 26]. The impact of temozolomide monotherapy in patients with BM and neuroendocrine neoplasms remains unclear. The current guidelines of BM from solid tumors recommend cytotoxic chemotherapy for chemosensitive tumors in patients with asymptomatic or small BM [27]. Since temozolomide is an active drug in proliferating neuroendocrine tumors and can penetrate the blood-brain barrier, it is a valuable option besides surgery and radiation.

Our study is limited in several ways. There are inherent limitations in retrospective analyses. The presented study group is inhomogeneous and includes many different primary tumor localizations. The primary tumor localization itself has prognostic effects and also influences metastasis. On the other hand, neuroendocrine neoplasms can occur ubiquitously and the more exact pathological classifications allow a better characterization of this entity only since a few years. In addition, the study cohort integrates 2 local databases, which does not allow statistical statements on incidence and prevalence of brain metastases. Despite the recording of brain metastases, the process of therapy decision can no longer be traced. This also includes the selection of the available therapy modalities. In addition, response and duration of the response of the brain metastases to specific therapies and their influence on survival cannot be evaluated retrospectively in a proper way.

## Conclusion

Although BM from NEN occur rarely, their appearance impair the prognosis significantly. In our series, grade 3 tumors, male gender and age above 60 years were poor prognostic indicators. Routine metastatic assessment of the brain could be implemented for patients with NEC and lung carcinoids, whereas it seems not to be justified in NETs of other origin. Generally, no treatment recommendations for BM can be drawn based on our data and the given literature. Further studies are mandatory to better define the diagnostic value of routine brain imaging and the treatment modalities in this subset of patients.

## Abbreviations

BM: brain metastases
CI: confidence interval
CTx: chemotherapy
G: grading
GEP-NEN: gastroenteropancreatic neuroendocrine neoplasms
mOS: median overall survival
mPFS: median progression-free survival
NEC: neuroendocrine carcinoma
NEN: neuroendocrine neoplasm
NET: neuroendocrine tumor
WBRT: whole brain radiotherapy
